# Amelioration of Alzheimer’s Disease Pathology in Zebrafish by Photobiomodulation

**DOI:** 10.3390/biomedicines13123121

**Published:** 2025-12-18

**Authors:** Binnur Eroglu, Daniela Velez, Kimya Jones, Ferenc Deak, Ali Eroglu

**Affiliations:** 1 Department of Neuroscience and Regenerative Medicine, Medical College of Georgia, Augusta University, Augusta, GA 30912, USA; 2 Department of Pathology, Medical College of Georgia, Augusta University, Augusta, GA 30912, USA; 3 Department of Obstetrics and Gynecology, Medical College of Georgia, Augusta University, Augusta, GA 30912, USA

**Keywords:** Alzheimer’s disease, neurodegenerative diseases, photobiomodulation, therapy, low-level laser therapy, zebrafish, okadaic acid

## Abstract

**Background/Objectives**: The zebrafish is a widely used research model due to its characteristics, such as being transparent during development, sharing 70% of its genes with humans, and having conserved features of vertebrate aging, including deterioration of mitochondrial and cognitive functions. While affecting approximately 15% of the world population, neurodegenerative diseases, such as Alzheimer’s disease (AD), are currently incurable, requiring testing of alternative treatment strategies. Hence, this study was conducted to test the hypothesis that an optimized photobiomodulation (PBM) therapy improves AD pathology through its multifaceted beneficial effects, including enhancing mitochondrial function and reducing oxidative stress and neuroinflammation. **Methods**: A pharmacological zebrafish model of AD was developed by adding small amounts (100 nM) of okadaic acid (OKA) directly to fish tanks for nine days. Next, some of OKA-treated and control zebrafish were subjected to an optimized near-infrared PBM therapy while others remain untreated. **Results**: When examined after OKA treatment, zebrafish brains displayed histological hallmarks of AD including, neurofibrillary tangles, vacuoles, and neuroinflammation. Behavioral tests using a T-maze revealed that OKA-treated zebrafish spent significantly less time in the reward arm than untreated controls (15.2% vs. 50%). In contrast, a sequential PBM therapy significantly reduced formation of neurofibrillary tangles, vacuoles, neuroinflammation, and improved mitochondrial biogenesis in brains of OKA-treated zebrafish while also improving their cognitive function as evidenced by being able to recall the reward arm and spending more time there similar to controls (55 and 57%, respectively). **Conclusions**: These findings suggest that (1) a fast, cost-effective zebrafish AD model can be developed using OKA treatment and (2) PBM therapy holds promise to ameliorate AD pathology.

## 1. Introduction

Short-lived invertebrate models, such as yeast (Saccharomyces cerevisiae), worms (Caenorhabditis elegans), and fruit flies (Drosophila melanogaster), have been extensively used in aging research and have helped reveal conserved pathways like the mammalian target of rapamycin and insulin/insulin-like growth factors [1]. However, these short-lived invertebrates cannot faithfully model some important aspects of human aging and related conditions, such as blood, bones, the adaptive immune system, and vertebrate-specific genes (e.g., APOE and p15INK4B). These limitations of invertebrate models can be overcome by using zebrafish (*Danio rerio*), a widely used vertebrate model system [2,3,4]. The zebrafish offers several advantages, including having a short generation time (3–5 months), a highly conserved genome sharing 70% of genes with humans, amenability to gene editing, a small size allowing high-throughput screening, and cost-effective housing and maintenance, while being transparent during development. Moreover, the zebrafish displays conserved features of vertebrate aging, such as spinal deformation due to muscle abnormalities, loss of vision, cognitive decline, and reduced mitochondrial function [5,6,7]. Overall, the zebrafish represents a cost-effective and efficient model system for aging-related human diseases and the development of effective therapies.

Neurodegenerative diseases, including Alzheimer’s disease (AD), Parkinson’s disease, Huntington’s disease, amyotrophic lateral sclerosis, and multiple sclerosis, are linked to aging and characterized by progressive neuronal loss and impaired nervous system functions [8]. According to projections of the World Health Organization and Alzheimer’s Disease International, neurodegenerative diseases will be the second leading cause of death after cardiovascular diseases by 2040, while the number of cases will increase to around 135 million by mid-century [9]. Among the neurodegenerative diseases, AD is the most prominent one and is characterized by primary hallmarks of extracellular amyloid-beta (Aβ) plaques and intracellular tau neurofibrillary tangles (NFTs) [10]. Although the underlying mechanisms behind AD are not yet fully understood, it is widely accepted that the development of the disease involves a series of detrimental events, including the formation of excessive Aβ plaques and intracellular NFTs, oxidative stress, neuroinflammation, and mitochondrial dysfunction, leading to progressive neuronal loss [11,12,13]. Until recently, the drugs approved by the Food and Drug Administration (FDA) for the treatment of AD afforded temporary relief by slowing disease progression and improving some symptoms [8,14]. While newly FDA-approved anti-Aβ antibodies may reduce the burden of Aβ plaques in the brain [15,16,17,18], readily achievable cures still remain elusive, requiring alternative treatment strategies. Hence, the objective of this study was two-fold: (1) establishing a pharmacological model of AD by treating zebrafish with okadaic acid, and (2) ameliorating AD pathology and cognitive impairment in the zebrafish model using an optimized near-infrared (NIR) photobiomodulation (PBM) therapy.

Okadaic acid (OKA) is a selective and potent inhibitor of protein phosphatase 2A (PP2A) [19,20]. By inhibiting PP2A, which dephosphorylates tau [21], OKA induces hyperphosphorylation of tau, leading to the formation of AD-driving toxic intracellular NFTs by detaching tau from microtubules and causing it to clump together. Rats and zebrafish treated with OKA develop distinct hallmarks of AD, which include β-amyloid plaques, tau aggregates/tangles, increased expression of kinase glycogen synthase 3β (GSK-3β), and impairment of learning and memory [20,22,23,24]. Upon addition to fish water, OKA is taken up by the gills and skin of zebrafish and transported to the brain [25]. Therefore, OKA treatment was selected to establish a zebrafish model of AD.

PBM, also known as low-level laser therapy (LLLT), is a promising therapeutic approach applicable to various medical conditions. Published studies show that PBM therapy ameliorates wound healing [26,27,28,29,30,31], arthritis [32], hypoxic–ischemic injury [33], stroke [34], and pain [35] while having anti-inflammatory effects [36]. PBM works through chromophores present in cells, notably in mitochondrial membranes [37,38]. Upon absorbing photons, such chromophores induce various signaling pathways depending on different wavelengths [39,40]. Specifically, wavelengths in the near-infrared (NIR) range activate mitochondria through releasing nitric oxide (NO) from Cytochrome C Oxidase (CCO), resulting in increased mitochondrial membrane potential [37,41] and thus in increased production of adenosine triphosphate (ATP) [38,42,43] and enhanced cell proliferation [41,42,44]. Our recent study also revealed that an optimized sequential PBM treatment in the NIR range improves mitochondrial function and reverses the aging of mesenchymal stem cells isolated from 2-year-old mice [45]. Based on these positive findings, the present study was conducted to test the hypothesis that an optimized NIR PBM treatment strategy can block the progression of AD and improve AD pathology and cognitive impairment by activating several signaling pathways, leading to improved mitochondrial function and suppression of neuroinflammation, and thus to amelioration of cellular health and neurogenesis.

## 2. Materials and Methods

### 2.1. Zebrafish Maintenance

All animal use protocols and proposed experiments have been approved by the Institutional Animal Care and Use Committee (IACUC, Protocol #2017-0882) and the Institutional Biosafety Committee (BSP#0610). Zebrafish were maintained by the Augusta University Transgenic Zebrafish Core in a temperature-controlled room at 28 °C under a light/dark cycle of 14 h of light and 10 h of darkness. Zebrafish were fed a standard diet of brine shrimp (Artemia salina) and TetraMin^®^ flakes (Tetra GmbH, Melle, Germany) twice daily.

### 2.2. Chemicals/Reagents

Unless otherwise stated, all chemicals and reagents were purchased from Thermo Fisher Scientific (Waltham, MA, USA). Thioflavin-S (#S10185) was obtained from TriStains (Dawn Scientific, Inc., Metuchen, NJ, USA). Silver nitrate (#S-6506) was purchased from Sigma-Aldrich (St. Louis, MO, USA). CF594 TUNEL Apoptosis Detection Kit (#30064) was acquired from Biotium (Hayward, CA, USA). A stock solution of tricaine methanesulfonate (#MS222, Syndel, Ferndale, WA, USA) was prepared in ultrapure water at a 4 mg/mL concentration, and its pH was adjusted to 7. The stock was diluted with fish water to prepare solutions for either anesthesia (0.2 mg/mL) or euthanasia (0.6 mg/mL).

### 2.3. Experimental Design

**Figure 1:**
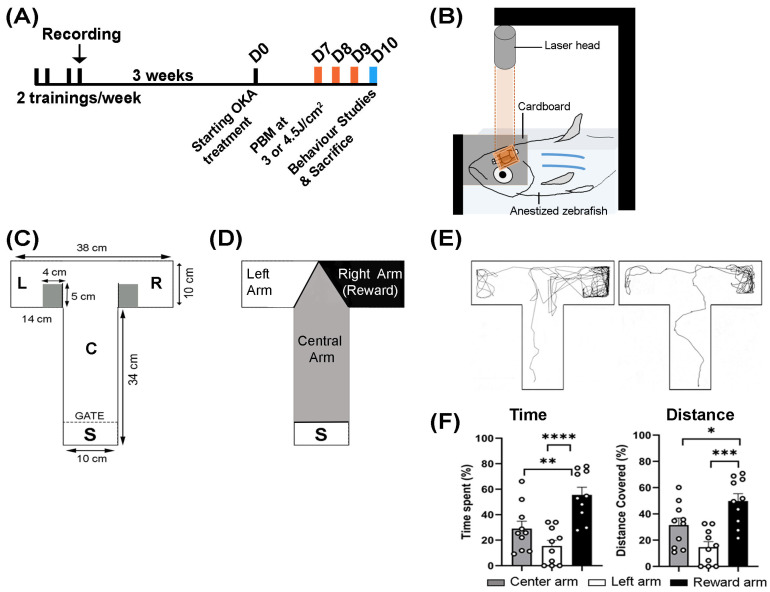
Experimental overview. (**A**) Schematic timeline of experimental interventions. Adult zebrafish were trained twice weekly for two weeks (black bars), with their final training session recorded (black arrow). After a three-week rest period, the fish were randomly allocated to the control and experimental groups. OKA treatment lasted nine consecutive days. PBM treatments were applied on days 7, 8, and 9 (orange bars). Behavioral tests were conducted on Day 10 (blue bar). (**B**) Schematic setup of the PBM treatment. An anesthetized zebrafish was positioned under a stabilized cardboard with an aperture of 2 mm by 3 mm to target the brain with a laser beam. Depending on the group, PBM at 3 or 4.5 J/cm^2^ was applied for three days in a row. The untreated controls underwent the same procedure, but the laser was off. (**C**) Schematic of the T-maze showing its dimensions: start chamber (S) where fish were held for initial adaptation, central arm (C), left arm (L), and right reward arm (R). (**D**) Diagram of the T-maze showing color-coded zones used by the TrackAnalyzer module to quantify time spent and distance covered in each zone. The right reward arm (black) was associated with food. (**E**) Two representative tracking plots recorded at the end of the learning/training phase (three weeks before drug treatment) display typical zebrafish behavior. (**F**) Quantification of all recorded plots showing the percentage of time spent and distance covered in each zone. The bar graphs show means ± s.e.m while open circles display individual data points. The tracking plots of ten randomly selected fish were analyzed. * *p* < 0.05, ** *p* < 0.01, *** *p* < 0.001, **** *p* < 0.0001.

The experimental overview is shown in Figure 1A. To perform behavioral studies on the effects of OKA and PBM treatments, adult zebrafish (~7 months old) were subjected to training/learning tasks in a T-maze setup (Figure 1A) for 2 weeks, with the final training session was recorded. After a three-week rest period, the zebrafish were randomly distributed into 6 groups: (1) vehicle control, (2) OKA treatment, (3) vehicle control with PBM at 3.0 J/cm^2^, (4) vehicle control with PBM at 4.5 J/cm^2^, (5) OKA treatment with PBM at 3.0 J/cm^2^, and (6) OKA treatment with PBM at 4.5 J/cm^2^. After the OKA and PBM treatments, behavioral tests were repeated. Each group received at least 4 females and 4 males.

### 2.4. Okadaic Acid Treatment

Okadaic acid Na salt (OKA, cat #O-5857, LC Laboratories, Woburn, MA, USA) was initially dissolved in 95% ethyl alcohol to prepare a 1 mM stock solution, which was then diluted with fish water to achieve a treatment concentration of 100 nM. An equivalent volume of ethyl alcohol was added to the control group as a vehicle control. The OKA treatment lasted nine consecutive days, with the fish water refreshed every other day. Zebrafish underwent behavioral tests before and after the OKA treatment.

### 2.5. Photobiomodulation (PBM) Treatment

To test the effectiveness of PBM in improving AD pathology, zebrafish were exposed to near-infrared (NIR) light at a continuous wavelength of 808 nm, emitted by a diode laser (Diode IR Laser System, MDL-III-808-1W, Dragon Lasers, Changchun, China). To calibrate and determine the appropriate power density, the head of a euthanized zebrafish was exposed to laser light at different intensities while measuring photon penetration using a sensor of a Coherent power/energy meter (Model FM 33-0506, Coherent Inc., Wilsonville, OR, USA) placed underneath the zebrafish skull. The measured values were used to adjust the fluence to desired doses, expressed as Joules/cm^2^ and calculated by multiplying the total irradiation time in seconds by the power output (W/cm^2^)

Before the PBM treatment, a zebrafish was anesthetized with tricaine in fish water and then placed into a slit on a sponge that was positioned in a plastic container filled with fish water to keep the fish wet and in the correct orientation. To target the brain, the sponge was placed in a pre-set position underneath a stabilized cardboard with a 2 mm × 3 mm opening, such that the laser beam was aiming at a 0.06-cm^2^ rectangular spot 1 mm posterior to the eyes (Figure 1B). The vehicle control groups (3 and 4) and OKA-treated groups (5 and 6) received PBM treatments at 100 mW/cm^2^ for either 30 s or 45 s, resulting in energy densities of 3.0 and 4.5 J/cm^2^, respectively. The PBM treatment was applied for three consecutive days. The zebrafish in the untreated control group were also anesthetized and underwent the same procedures, except that the laser power was not turned on.

### 2.6. Behavioral Studies

As shown in Figure 1A,C, behavioral studies were conducted before OKA treatment (learning/training task) and after OKA/PBM treatment (memory task) using a T-maze apparatus. If a behavioral test followed anesthesia, an interval of at least 24 h was maintained between the two events to ensure that the zebrafish fully recovered from any anesthesia effects [46]. The T-maze consisted of a long arm and two short side arms of 34 cm and 14 cm, respectively. The long arm had a start chamber (S) with a barrier where each fish was held for a few minutes to allow initial adaptation (Figure 1C). The right-hand section (R) of the T-maze was the reward arm associated with food (Figure 1C,D). To induce a favorable response to food reward, zebrafish underwent diet restriction for 22–24 h before each session. The T-maze was pre-filled with fresh fish water at 28 °C. First, a single zebrafish was transferred into the start chamber. After the adaptation period, the gate was slowly opened, allowing the fish to swim freely into any arm of the T-maze. When the zebrafish entered the reward chamber, approximately 3 TetraMin flakes were provided as food. The T-maze was cleaned between trials, and the fish water was replaced. The learning/training task was repeated twice weekly with a 2-day interval, for a total of four sessions. The memory task was performed on day 10 after the drug and PBM treatments (Figure 1A).

### 2.7. Video Recording and Animal Tracking

The behavior of each zebrafish was tracked and recorded using a webcam (C925e Logitech HD 1080p, Logitech, Lausanne, Switzerland) placed above the T-maze and connected to a laptop. The AnimalTracker ImageJ/Fiji plugin (https://animaltracker.elte.hu/, 2 September 2022) was used to track the animals [47]. To analyze the recorded fish tracks, files were converted to AVI format at 30 frames per second (fps) using ImageJ. The AnimalTracker ImageJ plugin has three main modules (i.e., the Tracker, TrackAnalyzer, and ZoneDesigner modules) working together. The video recordings are processed in the Tracker module, which determines the subject’s XY coordinates in each frame. The TrackAnalyzer module is used to visualize the trajectories produced by the Tracker module, while the ZoneDesigner module helps analyze different zones. This study utilized the Radial maze example package within the Tracker module. The XY coordinates provided by the Tracker module were used by the TrackAnalyzer module to calculate the distance covered and the time spent within the three predefined zones of the central, left, and right arms (Figure 1D).

### 2.8. Histology and Immunohistochemistry

At the end of each experiment (on Day 10), the zebrafish were euthanized using tricaine, and the heads of two females and two males from each group were fixed in 4% paraformaldehyde (PFA). The fixed tissues were decalcified with 0.5M EDTA (pH 7.3) for three days. The tissues were then processed, embedded in paraffin, and sectioned at 6-µm thickness. The serial sections were placed on microscope slides and left to dry at room temperature. After removing the paraffin with xylene, the sections were rehydrated through decreasing alcohol concentrations. The tissue sections underwent staining using various methods, including hematoxylin and eosin (H&E), Thioflavin-S, TUNEL, and Bielschowsky’s silver staining. H&E staining was performed by Georgia Esoteric and Molecular Laboratory (GEM), LLC at Augusta University.

For Thioflavin S (Thi-S) staining, the rehydrated sections were stained in a 1% aqueous Thioflavin S staining solution (filtered through Whatman #1) for 8 min at room temperature in the dark. The sections were then differentiated in 80% ethanol 2 times for 3 min each, rinsed with water 3 times, and mounted using aqueous mounting media. Coverslips were placed and sealed with clear nail polish. Fluorescence images were captured using a Keyence microscope (BZ-X710, Keyence Corp., Itasca, IL, USA). After taking fluorescence images, the coverslips were removed, and the slides were re-stained with H&E using standard protocols. Bright-field images were then captured from the same area of the previously captured fluorescence images. To quantify intracellular fluorescence intensities, ImageJ software was used and individual cells were selected with a freeform selection tool. Next, the area, integrated density, and mean gray value were measured. To assess background fluorescence, a nearby region without fluorescence was chosen, and the same parameters were measured. The corrected total cell fluorescence (CTCF) was calculated using the following formula: CTCF = integrated density − (area of the selected cell × mean fluorescence of the background reading).

For Bielschowsky’s silver staining (BSS), the rehydrated sections were stained in a preheated 10% silver nitrate solution for 25 min at 37 °C. Color development and toning were performed as described previously [48]. After capturing images, the coverslips were removed, and the slides were re-stained with H&E using standard protocols. Bright-field images were then captured with a Keyence microscope (BZ-X710). The quantification of BSS was performed using ImageJ software after converting the RGB images to 8-bit grayscale. A threshold was set to a fixed value to distinguish positive NFTs from the background before measuring the area and the area fraction (%) for each view.

To detect cell apoptosis, terminal deoxynucleotidyl transferase dUTP nick end labeling (TUNEL) was conducted using the CF594 TUNEL Apoptosis Detection Kit according to the manufacturer’s protocol. Briefly, deparaffinized sections were treated with proteinase K (20 μg/mL) in phosphate-buffered saline (PBS) at room temperature for 30 min and then rinsed with PBS. After incubating in the TUNEL equilibration buffer for 5 min, the sections were treated with freshly prepared TUNEL reaction mixture as instructed and incubated for 2 h in a humidified chamber at 37 °C. Next, the tissue sections were thrice washed in PBS containing 0.1% Triton X-100 and 5 mg/mL BSA for 5 min before being counterstained with Hoechst 33342 (#H1399) and mounted. Fluorescence images were captured using a Keyence microscope (BZ-X710).

### 2.9. Real-Time Polymerase Chain Reaction (RT-PCR)

A quantitative PCR analysis was performed on zebrafish brains to assess the effects of PBM. On day 10, the zebrafish were humanely euthanized, and their brains were collected. Total RNA was extracted from the brains using the RNeasy Mini Kit (Qiagen, #74004, Hilde, Germany), and reverse transcription was carried out with the qScript cDNA Synthesis Kit (Quantabio, #95047-025, Beverly, MA, USA). Signals were detected using SsoAdvanced Universal SYBR Green Supermix (BioRad, #1725270, Hercules, CA, USA) and the CFX Opus 96 Real-Time PCR System (BioRad, #12011319, Hercules, CA, USA). The data were normalized against the housekeeping gene Ribosomal protein L13a (RPL13a). The primer sequences are listed in Table 1.

### 2.10. Statistical Analysis

Experimental data were analyzed by one-way ANOVA using GraphPad Prism 9.1.0 (GraphPad Software, Inc., San Diego, CA, USA). Data from both sexes were combined for statistical analysis. Differences between groups were considered significant when the *p*-value was less than 0.05.

## 3. Results

### 3.1. Behavioral Response to OKA and PBM Treatments

To assess the cognitive function of OKA-treated zebrafish and the effect of PBM, we conducted behavioral tests both before (learning/training task) and after (memory task) OKA/PBM treatments using a T-maze apparatus (Figure 1A,C). Zebrafish underwent learning and training sessions twice a week, with a 2-day interval between the sessions, totaling four sessions (Figure 1A). Behavioral assessments conducted after the two-week training period (and three weeks before OKA treatment) revealed that zebrafish eagerly explored the reward arm more than the other arms (Figure 1D–F). They spent significantly more time and traveled a greater distance in the reward arm, indicating a normal behavioral response following the training period (Figure 1E,F).

**Figure 2:**
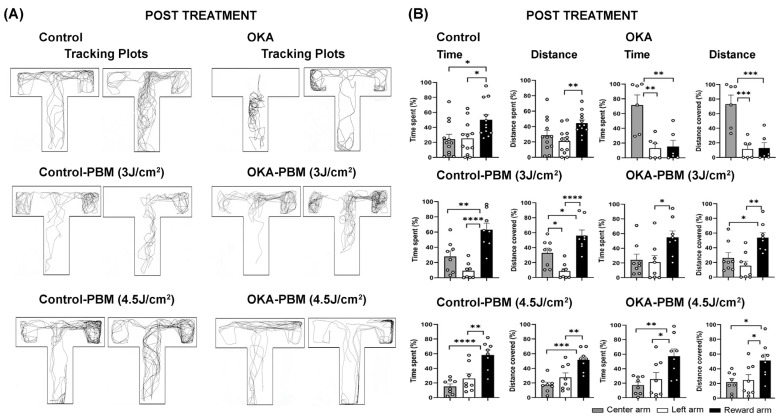
Behavioral response of zebrafish to OKA and PBM treatments on Day 10. (**A**) Representative tracking plots (two examples per group) showing the behavior of zebrafish in each group. (**B**) Quantification of the recorded tracking plots showing time spent and distance covered in each zone as percentages. The shaded, open, and solid black columns represent the central long arm, left arm, and right (reward) arm, respectively, as indicated by the color-coded zones in Figure 1D. The bar graphs show means ± s.e.m while open circles display individual data points. * *p* < 0.05, ** *p* < 0.01, *** *p* < 0.001, and **** *p* < 0.0001.

The memory task was conducted on day 10 following the culmination of the drug and PBM treatments. Vehicle-treated controls displayed behavior similar to that observed at the end of the learning/training phase, indicating retention of cognitive functions and memory (Figure 2A,B). In contrast, OKA-treated zebrafish spent significantly less time in the reward arm than controls (15.2% vs. 50%). After PBM treatment at both 3 J/cm2 and 4.5 J/cm2, OKA-treated fish showed improved cognitive function comparable to the controls, as evidenced by their ability to recall the reward arm and spend more time there (55 and 57%, respectively) (Figure 2A,B).

### 3.2. Effects of OKA and PBM Treatments on Intracellular Tau Aggregation

**Figure 3:**
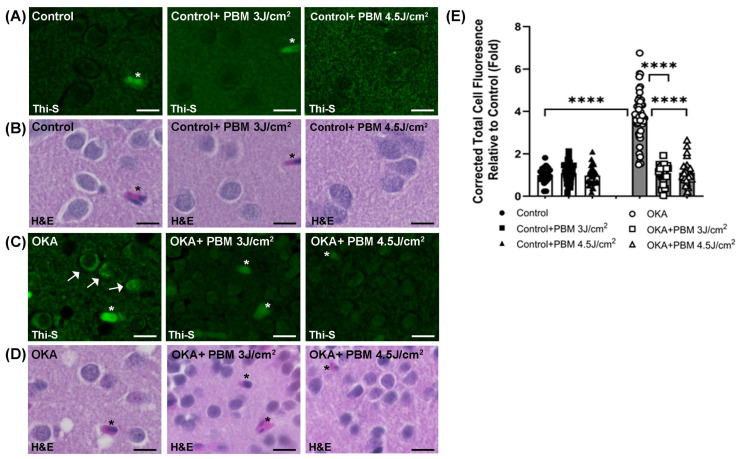
Thioflavin-S (Thi-S) staining of tau aggregates/neurofibrillary tangles in zebrafish brain sections. Shown are representative Thi-S stained (green) brain sections of control (**A**) and OKA-treated zebrafish (**C**), as well as their corresponding H&E stainings (**B**) and (**D**), respectively, where some intracellular tau aggregates/tangles and blood vessels are pointed out by arrows and asterisks, respectively. Scale bars: 10 μm. (**E**) The fold change of the corrected total cell fluorescence (CTCF) for each group relative to the untreated controls after quantification of Thioflavin-S fluorescence intensity by using ImageJ. Quantified only intracellular tau aggregates/tangles. The bar graphs show means ± s.e.m while open and solid signs display individual data points for the indicated groups. **** *p* < 0.0001.

The presence of intracellular tau aggregates/NFTs is a hallmark of AD and can be detected using Thioflavin-S (Thi-S) staining [53,54]. To better identify the Thi-S-stained structures in the brain sections, we re-stained the same sections with H&E (Figure 3B,D) as described in the Materials and Methods section. The H&E staining revealed that some of the structures nonspecifically stained by Thi-S were blood vessels (asterisks, Figure 3A–D).

No clear tau aggregates/tangles were observed in the brain sections of the untreated controls upon staining with Thioflavin-S (Figure 3A,E). In contrast, OKA treatment induced a significant increase in intracellular tau aggregates/NFTs (white arrows in Figure 3C; 1 vs. 3.98, *p* < 0.0001 in Figure 3E), whereas no such increase was evident after the sequential PBM treatment strategy at two different doses.

**Figure 4:**
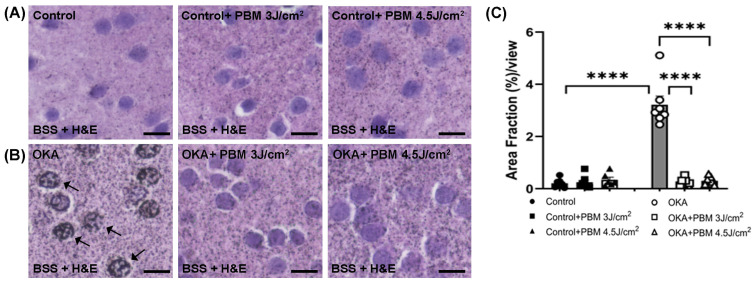
Bielschowsky’s silver staining (BSS) and H&E staining of tau aggregates/NFTs in zebrafish brain sections. Shown are representative brain sections of control (**A**) and OKA-treated zebrafish (**B**) where BSS was followed by H&E staining. Arrows indicate some intracellular tau aggregates/NFTs stained dark brown/black. Scale bars: 10 μm. (**C**) Shown is the amount of intracellular tau aggregates/NFTs after quantification of Area Fraction (%) per view using ImageJ. The bar graphs show means ± s.e.m while open and solid signs display individual data points for the indicated groups. **** *p* < 0.0001.

To further confirm and quantify the presence of intracellular tau aggregates/NFTs, we performed Bielschowsky silver staining (BSS) along with H&E staining (Figure 4A–C). The BSS also showed no clear intracellular tau aggregates/NFTs in the brain sections of untreated control zebrafish. By contrast, the brain sections of OKA-treated zebrafish exhibited many neurons with positive BSS, indicating the presence of tau aggregates/NFTs (Figure 4B, arrows), of which the area fraction was significantly greater than that of untreated controls (0.26 vs. 3.07, *p* < 0.0001; Figure 4C). Yet a sequential PBM treatment lowered the OKA-induced tau aggregate formation to a baseline level similar to that of untreated controls (Figure 4B,C).

Taken together, these results (Figure 3 and Figure 4) suggest an inhibitory/reversing effect of the NIR PBM treatment on the formation of tau aggregates/NFTs in OKA-treated zebrafish brains.

### 3.3. Effects of OKA and PBM Treatments on Brain Histology and Apoptosis

**Figure 5:**
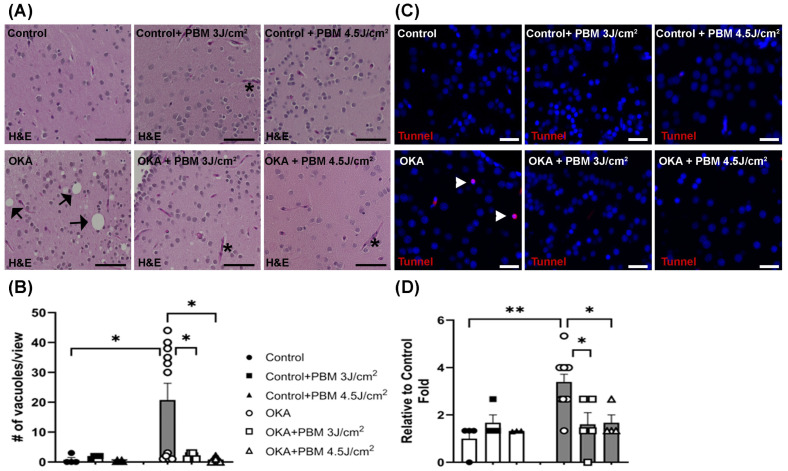
Hematoxylin and Eosin (H&E) and TUNEL staining of zebrafish brain sections. (**A**) Shown are representative brain sections of control and treated zebrafish, where some vacuoles and blood vessels are indicated by black arrows and asterisks, respectively. Scale bars: 50 μm. (**B**) The mean number of vacuoles per view. * *p* < 0.05. (**C**) Shown is TUNEL staining of representative brain sections of control and treated zebrafish, where some apoptotic cells (red) are labeled by white arrowheads. Cell nuclei were counterstained with Hoechst 33342 (blue). Scale bars: 20 μm. (**D**) The fold changes in apoptotic cells relative to the untreated control group. The bar graphs show means ± s.e.m while open and solid signs display individual data points for the indicated groups. * *p* < 0.05 and ** *p* < 0.01.

OKA is known to generate reactive oxygen species (ROS) and to induce cellular apoptosis [55]. To examine histological changes in the brains of control and treated zebrafish, their brain sections were stained with H&E. Compared to the vehicle-treated controls, zebrafish treated with OKA displayed an increased number of vacuoles in their H&E-stained brain sections, although the number of vacuoles varied considerably between individual fish (Figure 5A,B). These findings suggest that the OKA treatment used in this study increases neurodegeneration. Notably, the sequential PBM treatment strategy negated the neurodegenerative effect of the OKA treatment by significantly reducing the number of vacuoles.

Similarly, the TUNEL assay showed a significant increase in apoptotic cells following the OKA treatment, which was prevented/reversed by the sequential PBM treatment strategy (Figure 5C,D).

### 3.4. Effects of OKA and PBM Treatments on Neuroinflammation

**Figure 6:**
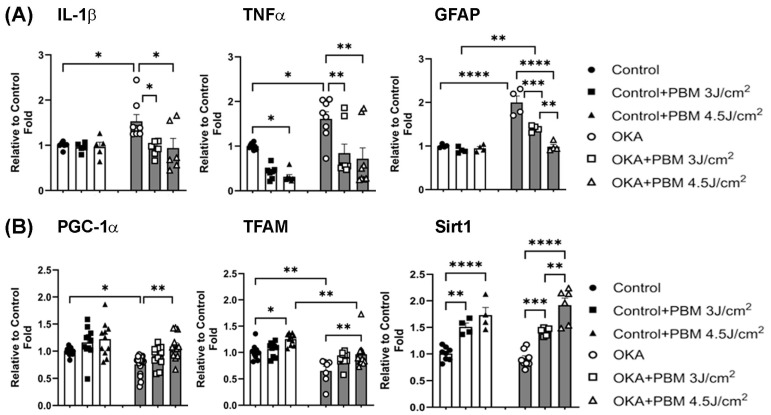
Effects of OKA and PBM treatments on expression of genes involved in neuroinflammation and mitochondrial biogenesis. (**A**) Relative mRNA levels of proinflammatory cytokines (IL-1ß and TNFα) and an astrocyte marker (GFAP) in zebrafish brain tissues analyzed by RT-PCR. (**B**) Relative mRNA expressions of PGC-1α, TFAM, and Sirt1 in zebrafish brain tissues analyzed by RT-PCR. The bar graphs show means ± s.e.m while open and solid signs display individual data points for the indicated groups. * *p* < 0.05, ** *p* < 0.01, *** *p* < 0.001, and **** *p* < 0.0001.

Neuroinflammation is another hallmark of AD [56]. Previous studies have shown that proinflammatory cytokines such as tumor necrosis factor-alpha (TNFα) and interleukin-1 beta (IL-1ß) are elevated in AD, contributing to neuroinflammation [57,58,59]. Astrogliosis is also a characteristic feature of AD pathology, where reactive astrocytes release proinflammatory cytokines and chemokines, further promoting neuroinflammation and neuronal cell death. Consistent with the pathogenesis of AD, it has been reported that OKA administration increases levels of IL-1ß and TNFα in the rat brain [60]. To examine how OKA and sequential PBM treatments affect neuroinflammation in the zebrafish brain, we analyzed expression of IL-1ß, TNFα, and glial fibrillary acidic protein (GFAP, an astrogliosis marker) genes in zebrafish brain samples from each group using quantitative RT-PCR. As shown in Figure 6A, these analyses revealed significantly increased expression of IL-1ß (1 vs. 1.53, *p* < 0.05), TNFα (1 vs. 1.61, *p* < 0.05), and GFAP (1 vs. 2, *p* < 0.0001) in OKA-treated zebrafish yet the expression levels of these proinflammatory cytokines and GFAP were comparable to those of untreated controls following the sequential PBM therapy. Overall, these findings suggest that the OKA treatment for nine days induces neuroinflammation and astrogliosis in the zebrafish brain, while the sequential PBM therapy is effective in blocking/reversing these neuroinflammatory events.

### 3.5. Effects of OKA and PBM Treatments on Mitochondrial Biogenesis

While several aspects of AD pathogenesis remain unclear, impaired mitochondrial biogenesis and function have been recognized as contributing factors [61]. Peroxisome proliferator-activated receptor gamma coactivator 1α (PGC-1α) and mitochondrial transcription factor A (TFAM) are key regulators of mitochondrial biogenesis and function [62,63,64]. It has been shown that expression of PGC-1α and TFAM is decreased in individuals with AD and in animal models of amyloidosis compared to healthy individuals [61,65,66]. It has also been reported that overexpression of PGC-1α reduces Aβ production, protects neural cells from oxidative stress-mediated death, and restores mitochondrial function under culture conditions [61,67,68]. Similarly, silent information regulator transcript 1 (Sirt1) is important in maintaining mitochondrial homeostasis [69]. Sirt1 levels are found significantly reduced in AD patients [70]. However, the precise effect of OKA on Sirt1 expression in the brain remains elusive. To examine how the OKA and sequential PBM treatments affect the expression of PGC-1α, TFAM, and Sirt1 and thus mitochondrial biogenesis, we analyzed the expression of these genes in brain samples of zebrafish from each group by quantitative RT-PCR. Our results revealed that compared to vehicle-treated controls, the OKA treatment significantly decreases expression of PGC-1α (1 vs. 0.75, *p* < 0.05) and TFAM (1 vs. 0.65, *p* < 0.01) while the sequential PBM treatment significantly improves expression of both genes (Figure 6B). Compared to the vehicle-treated control, the expression level of Sirt1 in OKA-treated animals was unaffected (1 vs. 0.9, non-significant). However, the sequential PBM therapy significantly increased Sirt1 expression in both control and OKA-treated animals (Figure 6B). Taken together, these results suggest that the PBM therapy exerts multifaceted beneficial effects.

## 4. Discussion

This study was conducted to test the hypothesis that a sequential NIR PBM treatment strategy, optimized earlier [45], can improve AD pathology induced by treating zebrafish with 100 nM OKA for 9 days. By comparatively assessing tau aggregates/tangles, neuroinflammation, mitochondrial biogenesis, and histological changes in brain sections of control and treated zebrafish, as well as by analyzing their behavioral responses, the present study shows that (1) the OKA treatment induces AD-like pathology in adult zebrafish, including impaired cognition/memory and (2) a sequential PBM treatment strategy greatly improves the OKA-induced AD pathology. These findings are of significance for the development of effective interventions against neurodegenerative diseases.

PBM is an emerging therapeutic modality shown to improve mitochondrial function [38,71], ATP production [42,43], cell proliferation [42,44,72,73], wound healing [26,31], arthritis [74], spinal cord injury [28], and traumatic brain injury [27]. Recently, our lab rejuvenated mesenchymal stem cells isolated from 2-year-old mice using an optimized sequential PBM treatment strategy [45]. The present study employed the same approach to ameliorate the OKA-induced AD-like pathology. Overall, the beneficial effect of PBM observed in this study aligns with the findings of the cited studies, although the underlying mechanism still needs further clarification.

PBM relies on the activation of chromophores (photoreceptors) by photons [75]. In mammalian cells, such chromophores are mainly found in mitochondrial membranes [37,38]. Mitochondrial cytochrome c oxidase (CCO) acts as a chromophore by absorbing NIR light [76,77]. When exposed to NIR light, mitochondrial CCO seems to release inhibitory nitric oxide (NO), which leads to the restoration of electron transport and thus to an increase in mitochondrial membrane potential and ATP production [38,42]. These events, along with secondary messengers, seem to activate various signaling pathways such as PI3K/AKT, RAS/ERK, PKC, and PKA/Sirt1/PGC-1α, resulting in the induction of antioxidants [38], suppression of inflammation and apoptosis [71,78,79], and enhancement of mitochondrial biogenesis [80,81] and cell survival/health [38]. Our findings on the multifaceted beneficial effects of PBM in diminishing the OKA-induced increase in expression of proinflammatory cytokines and astrogliosis, as well as in enhancing the expression of PGC-1α, TFAM, and Sirt1 (mitochondrial biogenesis and function), are in agreement with the above-cited studies and support simultaneous activation of multiple signaling pathways by the NIR PBM therapy.

Reactive astrocytes can form two distinct phenotypes (i.e., A1 and A2) in response to injuries and diseases [82]. The A1 phenotype produces proinflammatory cytokines and can induce neurotoxicity, while the A2 phenotype is neuroprotective and promotes neural recovery by increasing expression of neurotrophic factors and anti-inflammatory mediators, which, in turn, may facilitate phagocytosis and clearance of pathological Aβ plaques and tau tangles [83,84]. A shift from the neurotoxic A1 to the neuroprotective A2 phenotype may offer a promising therapeutic opportunity. It has been shown that PBM can promote this positive transformation of reactive astrocytes from A1 to A2 [85]. Our results also support these findings by showing decreased levels of proinflammatory cytokines and reduced astrogliosis after PBM treatment.

PBM is known to induce a biphasic inverted U-shaped dose response, with low and high doses being less effective [86]. In the present study, two different doses (3 J/cm^2^ and 4.5 J/cm^2^) were tested based on our previous experience with different cell types. Although both doses yielded satisfactory and similar results, a systematic testing needs to be performed in future studies to identify the optimal therapeutic dose.

As a vertebrate model organism, zebrafish offer several advantages, as explained in the Introduction section. In terms of neurodegenerative diseases, the zebrafish and human brains share many similarities. Consequently, the zebrafish is increasingly used in AD research [87]. The findings of our study suggest that a fast and cost-effective vertebrate model of AD can be established using zebrafish and small amounts (100 nM) of OKA for 9 days. Similar results reported by other studies [22,23,88,89,90,91,92,93,94] using different animal and cell models further support the use of OKA-based model systems in AD research. Collectively, OKA administration has been shown to cause oxidative stress, neuroinflammation, neurodegeneration, hyperphosphorylation of tau leading to intracellular tau tangles, Aβ plaques, and cognitive impairment. Hence, OKA-based model systems recapitulate various aspects of AD, although each model system has some shortcomings. For example, the zebrafish brain seems to have a greater regenerative capacity than the mammalian brain [95,96], which may have contributed to the remarkable beneficial effect of the PBM treatment observed in the present study.

Our results involving tau aggregates/tangles indicate that the sequential NIR PBM therapy inhibits and possibly reverses OKA-induced tau aggregates/tangles. Since the clearance of existing tau tangles usually takes a long time in mammals, these results were unexpected. Nevertheless, recent studies suggest that removal of tau tangles could be accomplished in some cases within a short period [97,98]. It is also possible that the higher regenerative capacity of the zebrafish brain may facilitate fast clearance of tau tangles. Another possibility is that quickly formed tau aggregates/tangles might be different from slowly formed ones in terms of allowing fast clearance. It should also be noted that unlike some murine studies, our NIR PBM therapy targeted the whole brain due to the smaller size of the zebrafish, which possibly contributed to the enhanced overall effect. Further research is needed to address the underlying mechanism.

The increased vacuolation and apoptosis observed in the brain sections of OKA-treated zebrafish in the present study are consistent with the findings of previous studies showing extensive vacuolation (spongiosis) and apoptosis in OKA-injected rat brains [88,99]. However, we noticed considerable variation in vacuole formation among the OKA-treated zebrafish. To overcome the blood-brain-barrier (BBB), rodent studies typically use intracerebroventricular (ICV) injection of OKA, which allows precise dosing and delivery of OKA directly to the target site. The BBB of the zebrafish is similar to mammalian counterparts but more permeable, allowing crossing of OKA [100]. Although this simplifies its delivery, the OKA uptake from fish water through gills and skin might be less precise and vary between the fish. This may explain the observed variation in vacuole formation among the OKA-treated zebrafish.

Previous behavioral studies demonstrated the functional learning and memory ability of zebrafish [100,101,102]. Our findings on the learning and memory tasks of zebrafish are in agreement with the cited studies. Likewise, our behavioral experiments showing memory deficit in OKA-treated zebrafish are in line with published studies and hallmarks of AD [22,93]. Moreover, our results showing that the sequential NIR PBM treatment prevents/reverses the OKA-induced memory deficit are also in accord with the beneficial effects of our PBM treatment strategy on the amelioration of AD pathology at the histological and molecular levels.

**Figure 7:**
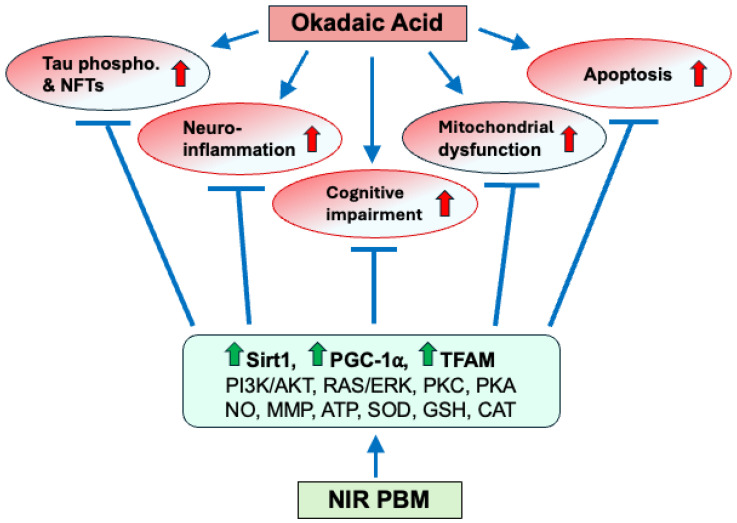
Schematic representation of the OKA-induced AD-like pathology and its amelioration by NIR PBM therapy. By selectively inhibiting PP2A, OKA induces hyperphosphorylation of tau and thus its detachment from microtubules, resulting in the formation of toxic tau aggregates and NFTs. Furthermore, our results indicate that OKA induces neuroinflammation, mitochondrial dysfunction, and apoptosis, all together leading to cognitive impairment and AD-like pathology. In contrast, a sequential NIR PBM therapy enhances mitochondrial biogenesis/function, antioxidant and anti-inflammatory defense systems, and pro-survival/cell-proliferation signals by increasing expression of Sirt1, PGC-1α, and TFAM, improving mitochondrial membrane potential (MMP), and increasing production of NO, ATP, antioxidants (e.g., SOD, GSH, CAT), as well as by activating PI3K/AKT, RAS/ERK, PKC, and PKA/Sirt1/PGC-1α signaling pathways. The findings of the present study are depicted as upward red (AD-inducing) and green (AD-ameliorating) arrows.

Based on the findings of the present study and published data, a working model is presented in Figure 7, proposing that the OKA treatment leads to (1) cognitive impairment/AD-like pathology by inducing (2) tau phosphorylation and NFTs, (3) neuroinflammation, (4) mitochondrial dysfunction, and (5) apoptosis. Meanwhile, NIR PBM counteracts the OKA-induced pathological changes by restoring the mitochondrial electron transport chain, leading to an increase in the mitochondrial membrane potential, adenosine triphosphate (ATP) production [42,43] and expression of Sirt1, PGC-1α, and TFAM, as well as by activating the PI3K/AKT, RAS/ERK, PKC, and PKA/Sirt1/PGC-1α signaling pathways, resulting in induction of antioxidants [38], suppression of inflammation and apoptosis [71,78,79], and enhancement of mitochondrial biogenesis/function [80,81] and cell survival [38]. The OKA-induced Aβ plaque formation is omitted in the current model due to lack of clear Aβ plaques in the zebrafish brain sections stained with Thioflavin-S and Bielschowsky’s silver staining. This should be addressed by other staining methods in future studies.

In conclusion, OKA is a useful tool to quickly generate a pharmacological zebrafish model of AD. As evidenced by its effectiveness in ameliorating tau aggregates/tangles, neuroinflammation, neurodegeneration, and mitochondrial biogenesis, as well as in restoring learning and memory, PBM is a promising therapeutic modality for neurodegenerative diseases. Further research is needed to fully realize the therapeutic potential of NIR PBM for the treatment of neurodegenerative diseases.

## Figures and Tables

**Table 1:** Primer pairs used for gene expression analysis by RT-PCR.

Name	Forward Primer (5′-3′)	Reverse Primer (5′-3′)
PGC1α [49]	TGAGGAAAATGAGGCCAACT	AGCTTCTTCAGCAGGGAAGG
TFAM	CGAAAGATTGCCCAGCAGT	GTCGTTTTTCCTCCGCAAA
GFAP [50]	ACCCGTGACGGAGAGATCAT	GCCAGTGTCTGAGCCTCATT
IL1ß [51]	CGGGCAATATGAAGTCACC	GTCCACATCTCCAGCCTGA
RPL13a [50]	TCTGGAGGACTGTAAGAGGTATGC	AGACGCACAATCTTGAGAGCAG
Sirt1 [52]	CCAAACGAAAGAAACGCAAAGA	CACAGGAAACAGACACCCCAG
TNFα [51]	TAGAACAACCCAGCAAACTC	TCTCCTTCTTCAACATCCAA

## Data Availability

The original contributions presented in this study are included in the article. Further inquiries can be directed to the corresponding author.

## Abbreviations

The following abbreviations are used in this manuscript:

Aβ	Amyloid beta
AD	Alzheimer’s Disease
ATP	Adenosine triphosphate
BSS	Bielschowsky’s silver staining
CAT	Catalase
CCO	Cytochrome C Oxidase
CTCF	Corrected total cell fluorescence
dUTP	Deoxyuridine triphosphate
GSK-3β	Glycogen synthase 3β
IACUC	Institutional Animal Care and Use Committee
EDTA	Ethylenediaminetetraacetic acid
ERK	Extracellular signal-regulated Kinase
H&E	Hematoxylin and eosin
GFAP	Glial fibrillary acidic protein
GSH	Reduced glutathione
IL-1β	Interleukin 1-beta
LLLT	Low-level laser therapy
MMP	Mitochondrial membrane potential
mTOR	Mechanistic Target of Rapamycin
NFTs	Neurofibrillary tangles
NIR	Near-infrared
NO	Nitric oxide
OKA	Okadaic acid
PBS	Phosphate-buffered saline
PGC-1α	Peroxisome proliferator-activated receptor gamma coactivator 1α
PFA	Paraformaldehyde
PI3K	Phosphoinositide 3-kinase
PBM	Photobiomodulation
PKA	Protein kinase A
PKC	Protein kinase C
PP2A	Protein Phosphatase 2A
ROS	Reactive oxygen species
RPL13a	Ribosomal protein L13a
RT-PCR	Real-time polymerase chain reaction
Sirt1	Silent Information Regulator Transcript 1
TFAM	Mitochondrial transcription factor A
Thi-S	Thioflavin-S
TNFα	Tumor necrosis factor-alpha
TUNEL	Terminal deoxynucleotidyl transferase dUTP Nick-End Labeling
